# Theranostic Potential of ^177^Lu-TLX591 with Best Standard-of-Care and ^68^Ga-PSMA-11 PET for Patients with Metastatic Castration-Resistant Prostate Cancer: Results from the Phase 1 ProstACT SELECT Trial

**DOI:** 10.3390/cancers18142331

**Published:** 2026-07-20

**Authors:** Nat Lenzo, Kenneth O’Byrne, Stanley Ngai, Laurence Krieger, Veronica Wong, David N. Cade

**Affiliations:** 1 GenesisCare, Alexandria, NSW 2015, Australia; 2 Medical Oncology, Princess Alexandra Hospital, Translational Research Institute, Queensland University of Technology, Woolloongabba, QLD 4102, Australia; 3 GenesisCare, St Leonards (North Shore Health Hub), St Leonards, NSW 2065, Australia; 4 Nepean Blue Mountains Local Health District Hospital, Penrith, NSW 2750, Australia; 5 Telix Pharmaceuticals, North Melbourne, VIC 3051, Australia

**Keywords:** prostate cancer, radiotheranostics, PSMA, antibody–drug conjugate, mCRPC, therapy, targeted radionuclide therapy, precision oncology, molecular imaging

## Abstract

**Background/Objectives**: Lutetium (^177^Lu) TLX591 (^177^Lu-TLX591) is a radio antibody–drug conjugate (rADC) that utilizes a novel monoclonal antibody-based approach for treatment of patients with prostate-specific membrane antigen (PSMA)-expressing metastatic castration-resistant prostate cancer (mCRPC). ProstACT SELECT (NCT04786847) was a multicenter, single-arm, open-label Phase 1 trial evaluating patient selection for ^177^Lu-TLX591 therapy using ^68^Ga-PSMA-11 PSMA positron emission tomography (PET) in a heterogenous sample of patients with mCRPC. Primary and key secondary objectives were to evaluate safety and tolerability, biodistribution, and organ radiation dosimetry of ^177^Lu-TLX591 in combination with the best standard of care (SOC) for patients with PSMA-expressing mCRPC who progressed despite prior treatment with an androgen receptor pathway inhibitor. **Methods**: Thirty patients received ^177^Lu-TLX591 intravenously in combination with investigator-determined SOC. Cohort 1 (*n* = 5) received an imaging dose of ^177^Lu-TLX591 (1 GBq [27 mCi]), followed 14 days later by one therapeutic dose (2.8 GBq [76 mCi]). Cohort 2 received two therapeutic doses of ^177^Lu-TLX591, 14 days apart. Baseline ^68^Ga-PSMA-11 PET was performed to confirm eligibility and was qualitatively compared with serial ^177^Lu-TLX591 single-photon emission computed tomography (SPECT)/CT, which was performed at five timepoints following the first ^177^Lu-TLX591 administration and also used to evaluate organ radiation dosimetry. Safety assessments included monitoring for treatment-emergent adverse events and collection of laboratory samples at specified timepoints used for biomarker analyses. **Results**: Tumor targeting observed on ^177^Lu-TLX591 SPECT/CT imaging was qualitatively consistent with uptake observed on ^68^Ga-PSMA-11 PET imaging. No new safety signals were observed. Radiation exposure was within safety limits, with the highest absorbed dose to liver (clearance organ; 2.44 ± 0.56 Gy/GBq) and with lower exposure to salivary glands (0.07 ± 0.03 Gy/GBq) and kidneys (0.64 ± 0.17 Gy/GBq). Activity was retained in tumor lesions through to the final protocol-specified imaging timepoint (312 h) following administration. **Conclusions**: In this Phase 1, single-arm study, ^68^Ga-PSMA-11 PET supported patient selection for ^177^Lu-TLX591 therapy. In a heterogenous population representative of a real-world setting, ^177^Lu-TLX591 therapy in combination with SOC demonstrated a manageable and predictable safety profile, durable retention, and low salivary gland radiation exposure. Further evaluation in larger, randomized studies is warranted.

## Simple Summary

Advanced prostate cancer remains difficult to treat, and current options can be hard on the body or may not stay in tumors long enough to work as well as hoped. This trial explores a new targeted radioactive treatment that may maintain quality of life and remain in tumors for longer than current treatments. This trial looked at whether imaging that shows the same target as the treatment target could help identify the right patients for this treatment and how the patients felt and what side effects they had when taking the treatment. The results suggest that the imaging did help find the right patients for the treatment, that the treatment stayed in the body for at least 2 weeks, and that most patients handled the drug well without long-lasting side effects; this supports the need for more research studies to continue to learn more about this treatment in more patients.

## 1. Introduction

Prostate cancer is the second most frequently diagnosed cancer in men globally and the leading cause of cancer deaths in men in more than 50 countries [1]. Compared with patients with non-metastatic disease, patients with metastatic disease experience an approximately 60% lower estimated 5-year survival [2], underscoring a substantial unmet need for effective treatments that both preserve quality of life and improve clinical outcomes. Current treatments for patients with castration-resistant prostate cancer (CRPC) and metastatic prostate cancer include androgen receptor pathway inhibitors (ARPIs) that inhibit androgen biosynthesis (e.g., abiraterone) and block androgen receptors (e.g., enzalutamide, apalutamide, and darolutamide); taxane-based chemotherapy (e.g., docetaxel and cabazitaxel) [3,4], which is associated with significant toxicity and often poorly tolerated, particularly in elderly or frail patients [5]; and immune checkpoint inhibitors (e.g., PD-1/PD-L1 inhibitors), which exhibit uncertain efficacy and potential adverse reactions [6].

In recent years, clinical care for patients with metastatic CRPC (mCRPC) has increasingly incorporated image-guided precision oncology targeting prostate-specific membrane antigen (PSMA), a transmembrane protein overexpressed in >95% of prostate cancer and minimally expressed in healthy tissue [7]. PSMA-targeted radioligand therapy (RLT) has broadened treatment options for patients with PSMA-expressing mCRPC, particularly following prior ARPI therapy [8]. The Phase 3 VISION trial demonstrated that ^68^Ga-PSMA-11 PET can support appropriate patient selection for PSMA-directed therapy [8]. Imaging-based confirmation of PSMA expression has since been incorporated into clinical practice guidelines and adopted as part of standard patient selection for PSMA-directed therapy [9,10,11]. Despite these advances, important limitations remain, potentially related to the small-molecule-based approach of currently available PSMA-targeted RLTs. Limitations include suboptimal efficacy in real-world settings, rapid systemic clearance that may limit tumor residence time, off-target radiation exposure to normal organs such as the salivary glands and kidneys that may contribute to xerostomia and nephrotoxicity [12,13], and time-intensive dosing schedules [14]. These considerations may become increasingly important as RLTs move earlier in the disease course, when patients may have longer life expectancies and greater cumulative risk from radiation exposure, renal toxicity, and treatment-related adverse events [15]. Accordingly, an unmet need exists for alternative PSMA-targeting strategies with distinct pharmacologic and biodistribution characteristics.

^177^Lu-TLX591 is a PSMA-targeting radio antibody–drug conjugate (rADC) that utilizes a monoclonal antibody-based approach. Monoclonal antibody-based PSMA-targeting may offer a differentiated approach for patients with mCRPC. Monoclonal antibodies are substantially larger molecules than small molecules, resulting in slower systemic clearance, prolonged circulation time, and a distinct biodistribution and safety profile. The larger molecular size may also limit access to certain normal tissues, including salivary glands, renal tubules, and the small intestine, potentially reducing off-target radiation exposure to organs commonly affected by small-molecule RLTs [16]. Plausibly, these pharmacologic characteristics may facilitate sustained tumor residence and prolonged engagement of PSMA-expressing lesions, supporting extended radiation delivery within the tumor microenvironment [17,18]. Previous Phase 1 and Phase 1/2 trials of ^177^Lu-TLX591 demonstrated a predictable safety and tolerability profile, particularly when fractionated dosing was utilized in patients with mCRPC [19,20,21], warranting continued research to better characterize its safety profile and pharmacologic properties in combination with standard-of-care therapies.

Reflecting recent regulatory and clinical emphasis on imaging-based confirmation of target expression before initiating targeted therapy, ProstACT SELECT (NCT04786847) was designed as a multicenter, single-arm, open-label trial to evaluate ^68^Ga-PSMA-11 positron emission tomography (PET) for patient selection before ^177^Lu-TLX591 therapy. Primary and key secondary objectives were to evaluate safety and tolerability, biodistribution, and organ radiation dosimetry of ^177^Lu-TLX591 in combination with best standard of care for patients with PSMA-expressing mCRPC who have progressed despite prior treatment with ARPIs.

## 2. Materials and Methods

### 2.1. Trial Design and Patients

ProstACT SELECT was a Phase 1 trial (NCT04786847) with 2 sequential cohorts. The 2-cohort design was used to characterize the biodistribution, organ dosimetry, and safety of ^177^Lu-TLX591 in a stepwise manner before broader evaluation of the intended therapeutic regimen. Cohort 1 (*n* = 5) received an initial sub-therapeutic imaging administration of ^177^Lu-TLX591 at tracer level of radioactivity (“imaging dose”; 1 GBq [27 mCi]) followed 14 days later by one therapeutic administration (“therapeutic dose”; 2.8 GBq [76 mCi]), enabling early characterization of whole-body distribution, source-organ time–activity behavior, and acute safety prior to repeat therapeutic dosing in combination with SOC. Following completion of dosimetry and safety assessments in Cohort 1, an independent data safety monitoring board (DSMB) reviewed biodistribution, dosimetry, and safety data. Progression to therapeutic-dose administration and enrollment of subsequent patients proceeded only after DSMB review and recommendation. Cohort 2 (*n* = 25) was designed to evaluate the intended clinical dosing regimen of two therapeutic doses of ^177^Lu-TLX591 administered 14 days apart in combination with SOC. Patients enrolled in Cohort 1 did not transition into Cohort 2.

Eligible patients included males aged ≥ 18 years with histologically confirmed adenocarcinoma of the prostate, evidence of metastatic disease, PSMA positivity confirmed by ^68^Ga-PSMA-11 PET, prior ARPI therapy (enzalutamide or abiraterone plus prednisone) for ≥12 weeks, Eastern Cooperative Oncology Group (ECOG) performance status of 0–2 with an estimated life expectancy of ≥6 months, testosterone levels < 50 ng/dL, and adequate organ function. PSMA positivity was defined as at least one site of metastatic disease with a radiolabeled intensity significantly greater than normal liver (maximum standardized uptake value [SUV_max_] at least 1.5 times SUV_max_ of normal liver) on ^68^Ga-PSMA-11 PET. Patients with prior PSMA-targeted therapy, recent systemic anti-cancer therapy or radiotherapy, uncontrolled pain, significant comorbidities, or other active malignancies were not eligible.

During trial participation, patients were permitted to receive the following: ARPIs and a bisphosphonate or denosumab regimen provided that the patient had proven tolerance (no changes were permitted throughout the duration of the trial, except in the event of patient intolerance or documented disease progression); supportive measures (pain medications, hydration, transfusions, growth factors for hematological support, etc.); ketoconazole; hormonal agents (single or combinations), estrogens including diethylstilbestrol (DES) and estradiol; luteinizing hormone-releasing hormone analogs for testosterone suppression; palliative external beam or radiation involving seeds (no systemic radiopharmaceuticals); bone-targeted agents (e.g., zoledronic acid, denosumab, any bisphosphonates) provided that the patient had been receiving and tolerating treatment for ≥30 days prior to enrollment.

Prior medications were recorded and defined as any medication that was started before ^177^Lu-TLX591 administration, regardless of when the medication was stopped.

### 2.2. Trial Treatments, Procedures, and Assessments

Patients were sequentially assigned to 1 of 2 cohorts. Patients in Cohort 1 received 1 GBq (27 mCi; imaging dose) ^177^Lu-TLX591 and 14 days later received 2.8 GBq (76 mCi; therapeutic dose) ^177^Lu-TLX591. Patients in Cohort 2 received 2 total therapeutic doses (2.8 GBq [76 mCi] ^177^Lu-TLX591 per dose), with each dose administered 14 days apart. ^177^Lu-TLX591 was administered as a slow intravenous infusion over 5 to 15 min. Best SOC was determined by the investigator in accordance with local clinical practice and could include a range of ongoing systemic therapies and supportive care measures, resulting in heterogeneity of concomitant treatments across the trial population.

Patients underwent serial SPECT/CT at 4 h, 24 h, 96 h, 168 h, and 312 h following ^177^Lu-TLX591 administration. Dosimetry analysis was performed in accordance with the MIRD schema, with time–activity data fitted using mono- or bi-exponential functions to derive time-integrated activity coefficients for each source organ. Organ absorbed doses and whole-body effective dose estimates were calculated using OLINDA/EXM V 2.2.3 (Hermes Medical Solutions; Stockholm, Sweden). Samples for biomarker analyses were taken on Days 168, 252, and 336 (long-term follow-up visits).

Biodistribution of ^177^Lu-TLX591 was qualitatively compared with baseline ^68^Ga-PSMA-11 PET. Centralized image review was conducted by experienced nuclear medicine physicians. All imaging acquisition, registration, segmentation, and dosimetry procedures were standardized and predefined prior to trial initiation.

Safety was evaluated by monitoring treatment-emergent adverse events (TEAEs), laboratory parameters, vital signs, physical examinations, and 12-lead electrocardiograms (ECGs). Safety assessments were conducted during the screening period (Day −35 to −14), treatment period (Days 0, 11, 13, and 14), short-term follow-up visits (Days 21, 28, 35, 42, 56, and 112), and long-term follow-up visits (Days 168, 252, and 336 and periodically thereafter). TEAEs were defined as events occurring from the time of first administration of ^177^Lu-TLX591 until the final follow-up visit and were collected continuously at all patient interactions.

TEAE incidence, frequency, severity, seriousness, and relationship to ^177^Lu-TLX591 were defined by the Medical Dictionary for Regulatory Activities (MedDRA) version 24.1 and graded according to the National Cancer Institute Common Terminology Criteria for Adverse Events (CTCAE) version 5.0. Symptomatic skeletal event (SSE) was defined as the use of external beam radiation to relieve bone pain, occurrence of a new symptomatic pathological fracture (vertebral or nonvertebral), occurrence of spinal cord compression, or tumor-related orthopedic surgical intervention [22].

Early indicative efficacy signals were assessed by radiographic progression-free survival (rPFS), objective response rate (ORR), and biochemical response as indicated by prostate-specific antigen (PSA), alkaline phosphatase (ALP), and lactate dehydrogenase (LDH) levels.

rPFS was defined as the time from enrollment to the first occurrence of radiographic disease progression or death from any cause, whichever occurred first. Disease progression was assessed by centralized independent review according to Response Evaluation Criteria in Solid Tumors (RECIST) version 1.1 for soft tissue lesions and Prostate Cancer Working Group 3 (PCWG3) criteria for bone lesions. Imaging assessments (CT, MRI, and ^99m^Tc bone scans) were performed at baseline, every 8 weeks for the first 24 weeks, and every 12 weeks thereafter until documented progression. Progression in bone was confirmed by a second scan at least 6 weeks after the initial finding, per PCWG3 recommendations. Patients without a documented progression event were censored at the date of last disease assessment.

ORR was defined as the proportion of patients achieving a complete response (CR) or partial response (PR) as their best overall response, based on RECIST v1.1 for measurable soft tissue disease. For patients with bone-only disease, response was assessed according to PCWG3 criteria.

### 2.3. Image Analysis and Dosimetry

Serial quantitative SPECT/CT was acquired approximately 4, 24, 96, 168 and 312 h after the first ^177^Lu-TLX591 administration. Imaging was performed on qualified and calibrated SPECT/CT systems using a medium-energy general-purpose collimator, ≥120 projections with 3° angular sampling, OSEM reconstruction, CT-based attenuation correction, triple-energy-window scatter correction, resolution recovery where available, and a 128 × 128 matrix. Patients were imaged from vertex to pelvis using 2–3 bed positions, and a ^177^Lu calibration source was included at each imaging timepoint for quantitative quality control.

Image analysis was performed centrally using MIM v7.2.8. Serial SPECT/CT datasets were deformably registered to the first post-treatment scan, and source-organ volumes of interest (kidneys, liver, lungs, spleen, salivary glands and bone marrow) were delineated on CT and propagated across timepoints. Organ activity was extracted from co-registered SPECT images, expressed as percentage injected dose, and fitted using mono- or bi-exponential models to derive time-integrated activity coefficients. Absorbed doses were calculated according to the MIRD schema using OLINDA/EXM v2.2.3 and ICRP 89 reference organ masses and are reported as Gy/GBq.

Tumor dosimetry was performed in Cohort 2 for up to five representative lesions per patient (SUVmax > 3). Lesions were contoured on baseline ^68^Ga-PSMA-11 PET using an SUV > 3 threshold and transferred to serial ^177^Lu-TLX591 SPECT/CT datasets following deformable registration. Lesion activity was quantified from co-registered SPECT images, and absorbed doses were calculated using the OLINDA/EXM unit-density sphere model. Bone lesions were corrected using density-weighted tumor S values (1.3 g/cm^3^). For lesions > 10 mL, absorbed dose was additionally estimated in a 10.3 mL lesion-core volume following partial-volume correction using site-specific recovery coefficients derived from IEC phantom measurements. Blood radioactivity concentration data were analyzed using a mono-exponential one-compartment model to derive pharmacokinetic parameters.

Quality control procedures were centrally implemented by an imaging core laboratory, including verification of protocol compliance, dataset completeness, image quality, and consistency between DICOM metadata and trial documentation. Standardization of acquisition and reconstruction parameters, reproducible patient positioning, and repeated calibration measures were required to ensure consistency across longitudinal imaging. Independent central review supported objective interpretation of imaging endpoints. Formal inter-observer variability analyses were not specified. To minimize interpretation bias, biodistribution comparisons and disease assessments were evaluated centrally by qualified readers using predefined imaging procedures, standardized acquisition and reconstruction parameters, and independent review processes for radiographic progression endpoints.

### 2.4. Statistical Analysis

A qualitative comparison of ^177^Lu-TLX591 SPECT/CT with ^68^Ga-PSMA-11 PET images was performed, evaluating tumor and non-tumor uptake. The comparison was descriptive and based on central image interpretation by appropriately qualified readers; no formal statistical analysis or quantitative comparison was planned or performed.

No formal sample-size calculation or hypothesis-testing framework was prespecified. The planned sample size was based on clinical and feasibility considerations and was considered sufficient to evaluate the primary objectives of biodistribution, organ dosimetry, safety, and tolerability. The trial was not powered to demonstrate conclusive efficacy outcomes, and all efficacy analyses were interpreted as exploratory and hypothesis-generating.

All statistical analyses were descriptive unless otherwise specified. Continuous variables were summarized using the number of non-missing observations, mean, standard deviation, coefficient of variation, median, interquartile range, minimum, and maximum. Categorical variables were summarized using frequencies and percentages, with the denominator defined as the number of patients in the relevant analysis population with non-missing data unless otherwise specified. Exact two-sided 95% confidence intervals (CIs) for objective response and PSA response rates were calculated using the Clopper–Pearson method. The Clopper–Pearson 95% CIs presented for PSA, ALP, and LDH were calculated by Microsoft Copilot Premium, using Python V3.12.9 with SciPy V1.13.1. For rPFS, patients without documented progression were censored at the date of the last adequate disease assessment; if progression was observed after two or more consecutive missed or inadequate assessments, censoring occurred at the date of the last adequate assessment prior to the missing interval, consistent with the prespecified statistical analysis plan. Analyses were performed and verified by an independent contract research organization according to the prespecified statistical analysis plan using SAS version 9.4.

**Figure 1:**
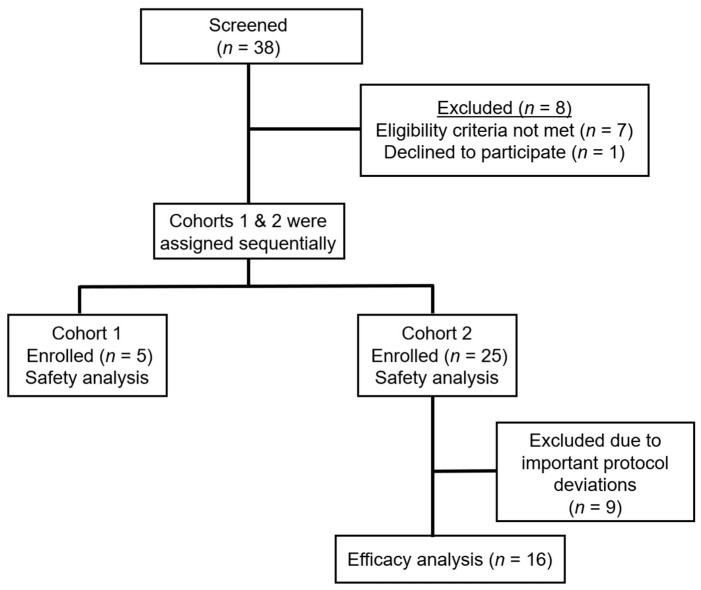
Patient enrollment. Cohorts 1 and 2 were enrolled sequentially. All patients in Cohorts 1 and 2 comprised the safety population. Of 25 patients enrolled in Cohort 2, 16 patients met protocol-defined criteria for inclusion in the efficacy analysis; 9 patients were excluded due to important protocol deviations.

Data from patients who received at least 1 administration of ^177^Lu-TLX591 were used for the safety analyses, including tolerability, treatment exposure, biodistribution, and dosimetry outcomes. This included Cohort 1 (*n* = 5) and Cohort 2 (*n* = 25). Following final database lock, data from 9 patients from Cohort 2 were excluded from the efficacy analysis as per protocol and statistical analysis plan due to important protocol deviations reported (Supplementary Table S1). Early indicative efficacy analyses (PSA response, serum changes in ALP, LDH) were conducted in Cohort 2 patients who received both therapeutic doses of ^177^Lu-TLX591 (*n* = 23). Efficacy analyses were repeated in the per-protocol population, defined as patients without important protocol deviations; this population included Cohort 2 patients who received both therapeutic doses and met prespecified per-protocol criteria (“efficacy population”; *n* = 16; Figure 1).

Organ absorbed doses, normalized cumulative radioactivity, and whole-body effective dose following ^177^Lu-TLX591 administration were summarized descriptively by organ and cohort. Safety outcomes, including treatment-emergent adverse events (TEAEs), serious adverse events, adverse events of special interest, adverse events leading to discontinuation or withdrawal, and laboratory abnormalities, were summarized descriptively by cohort and overall. Patients with multiple occurrences of the same adverse event were counted once for incidence summaries, using the maximum severity grade and most related causality category where applicable. Laboratory values were summarized as observed values and change from baseline at scheduled visits, with abnormal and clinically significant values summarized separately.

Time-to-event analyses, rPFS, time to a first symptomatic skeletal event (SSE), and overall survival (OS) were calculated using Kaplan–Meier methods. Median event times and corresponding 95% CIs were estimated where appropriate. rPFS was defined as the time from enrollment to radiographic disease progression by RECIST 1.1 for soft-tissue disease and/or PCWG3 criteria for bone disease or death from any cause, whichever occurred first. Patients without documented progression were censored at the date of last progression assessment. For overall survival, patients without documented death were censored at the date of data cutoff or last known contact, whichever occurred first. Patients who initiated subsequent anti-cancer therapy without prior documented disease progression were censored at the last tumor assessment before initiation of the new therapy. Time to first symptomatic skeletal event was defined as the time from first dosing to the first symptomatic skeletal event, with censoring at the end of trial or death, whichever occurred first.

Missing data were not imputed. Analyses were based on observed data, except where endpoint-specific rules were prespecified. Patients with unknown or missing RECIST response were treated as non-responders in response-rate analyses and included in the denominator. For prior and concomitant medications with missing or partially missing start or stop dates, medications were classified as both prior and concomitant when timing relative to trial treatment could not be determined.

## 3. Results

### 3.1. Patient Demographics and Prior Medications

A total of 38 patients were screened, of which 30 patients were enrolled and received at least one administration of ^177^Lu-TLX591 (Cohort 1, *n* = 5; Cohort 2, *n* = 25). Of the 25 patients in Cohort 2, 23 patients received two administrations of ^177^Lu-TLX591 (i.e., both therapeutic doses; Figure 1). The median age of all enrolled patients was 72.5 years (range 58–85), and 76.7% were Caucasian (Table 1).

All 30 patients received prior hormone antagonist and related agents (e.g., enzalutamide, abiraterone, abiraterone acetate, bicalutamide), and 23 patients (76.7%) received hormones and related agents (e.g., leuprorelin acetate, goserelin acetate, triptorelin acetate; Table 1). Cohort 2 was heterogeneous with regards to previous ARPI exposure: five patients (20.0%) had previously received docetaxel, while prior ARPI exposure included abiraterone (28.0%), enzalutamide (28.0%), or both agents (8.0%; Supplementary Table S2).

### 3.2. Safety

No significant abnormalities were observed in laboratory parameters, vital signs, physical examinations, and 12-lead ECGs. Of 212 total reported TEAEs in 30 total patients, 125 (59.0%) TEAEs were considered treatment-related, occurring in 25 (83.3%) patients. Of TEAEs considered treatment-related, fatigue was the most commonly reported non-hematological TEAE (19 events in 18 [60.0%] patients). Nausea and anemia were reported in five (16.6%) and six (20.0%) patients, respectively. Thrombocytopenia, neutropenia, lymphopenia, leukopenia, and anemia were reported in 14 (46.7%) patients, 10 (33.3%) patients, 7 (23.3%) patients, 6 (20.0%) patients, and 6 (20.0%) patients, respectively.

In Cohort 1 (*n* = 5), 15 total TEAEs were reported, of which four (26.7%) TEAEs were considered treatment-related, occurring in two (40.0%) patients. Grade 1 fatigue was the most commonly reported TEAE considered treatment-related (two events in two [40.0%] patients). Only one hematological TEAE considered treatment-related was reported (transient Grade 2 neutropenia). No Grade ≥ 3 TEAEs were reported in Cohort 1. SSE occurred in one (20.0%) patient.

**Figure 2:**
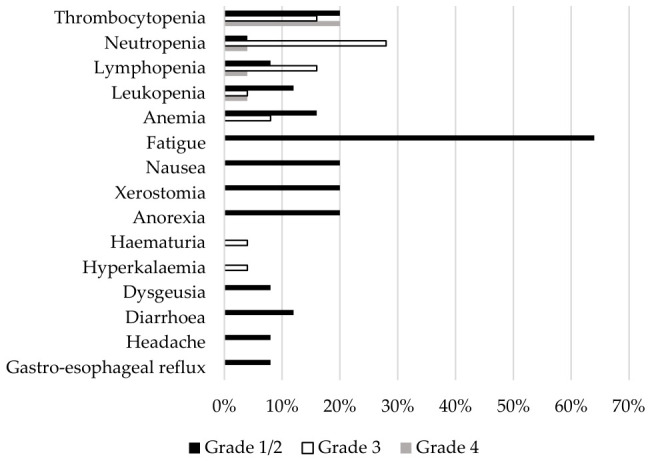
Summary of treatment-emergent adverse events considered treatment-related in Cohort 2 (*n* = 25 patients). Bars represent the proportion of patients who experienced each event by maximum CTCAE v5.0 grade: Grade 1/2 (black), Grade 3 (white), and Grade 4 (gray). Patients may have experienced more than 1 event or more than 1 grade of the same event. CTCAE: Common Terminology Criteria for Adverse Events.

In Cohort 2 (*n* = 25), a total of 197 TEAEs were reported, of which 121 (61.4%) TEAEs were considered treatment-related, occurring in 23 (92.0%) patients. Hematologic TEAEs were reported in 18 patients (72.0%). Of all patients in Cohort 2, the most common non-hematologic TEAEs considered treatment-related were fatigue (occurring in 16 [64.0%] patients) and nausea (occurring in five [20.0%] patients). Grade 1 xerostomia was reported in five (20.0%) patients; all events were transient. Grade 3 hematuria and hyperkalemia, considered related to ^177^Lu-TLX591, occurred in one (4.0%) patient each; both patients recovered to baseline. Other Grade ≤ 2 TEAEs considered treatment-related occurring in >1 patient included anorexia (five [20.0%] patients); diarrhea (three [12%] patients; and dysgeusia, headache, and gastro-esophageal reflux (two [8.0%] patients each) (Figure 2). The most common hematologic TEAEs considered treatment-related (any Grade) were thrombocytopenia (25 events in 14 [56.0%] patients) and neutropenia (11 events in 9 [36.0%] patients). Other hematologic TEAEs considered treatment-related included lymphopenia (seven events in six [24.0%] patients), leukopenia (six events in five [20.0%] patients), and anemia (six events in six [24.0%] patients) (Figure 2). Grade 4 thrombocytopenia, lymphopenia, and neutropenia occurred in five (20.0%) patients, one (4.0%) patient, and one (4.0%) patient, respectively (Figure 2). SSE occurred in four (16.0%) patients with a median time to SSE of 169 days.

Two patient deaths deemed unrelated to ^177^Lu-TLX591 occurred during the trial. One patient died due to intracerebral hemorrhage (Cohort 1), and one patient died due to disease progression (Cohort 2).

### 3.3. Organ Radiation Dosimetry and Biodistribution

The mean whole-body effective dose of ^177^Lu-TLX591 was 0.24 ± 0.04 Sv/GBq in Cohort 1 and 0.23 ± 0.02 Sv/GBq in Cohort 2. In Cohort 2, among all evaluated organs, the highest mean absorbed doses were observed in the liver (2.44 ± 0.56 Gy/GBq), spleen (0.99 ± 0.47 Gy/GBq), and kidneys (0.64 ± 0.17 Gy/GBq). The mean absorbed dose to red marrow, excluding patients with extensive bone infiltration, was 0.17 ± 0.04 Gy/GBq. Salivary gland exposure was low, with a mean absorbed dose of 0.07 ± 0.03 Gy/GBq (Table 2).

A total of 65 lesions were evaluated for participants in Cohort 2 (*n* = 25). Tumor sites include bone (*n* = 40), nodal (*n* = 23) and visceral (*n* = 2). The average tumor volume by site was 28.54 ± 30.31 mL for bone, 30.20 ± 36.39 mL for visceral and 31.60 ± 27.37 mL for nodal lesions. The mean absorbed dose for all tumors was 0.76 ± 0.57 Gy/GBq, with absorbed doses in bone, visceral and nodal lesions of 0.98 ± 0.59, 0.84 ± 0.81 and 0.36 ± 0.16 Gy/GBq, respectively. A summary of individual tumor dose after two cycles of 2.8 GBq is provided in Supplemental Figure S1.

Serial SPECT/CT imaging after the first dose of ^177^Lu-TLX591 showed that activity was retained in tumor lesions through to the final protocol-specified imaging timepoint, approximately 312 h (13 days) after administration. Estimated biological half-life of lesion was 326.02 h, and liver was 269.52 h.

**Figure 3:**
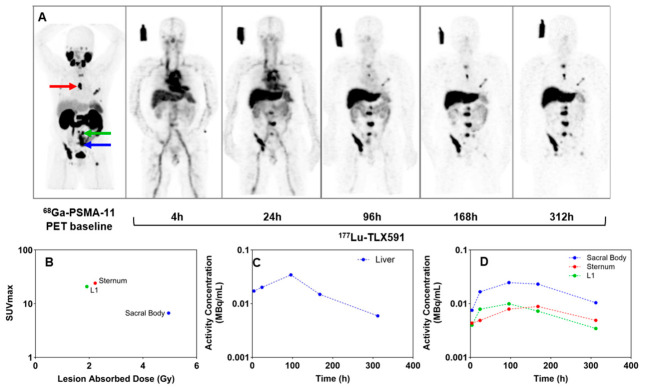
Representative patient imaging, SUV_max_, and activity concentration in liver and lesions. (**A**) Baseline ^68^Ga-PSMA-11 PET acquired at least 2 weeks prior to therapy noting the lesions L1 (green), sacrum (red) and sacral body (blue) used for dosimetry analysis. Serial SPECT/CT maximum-intensity projections were acquired at 4, 24, 96, 168, and 312 h after administration of the first cycle of ^177^Lu-TLX591. (**B**) Descriptive plot of SUV_max_ with lesion dose (Gy). Activity concentration (MBq/mL) in liver (**C**) as the main organ of clearance and lesions (**D**), illustrating sustained activity in skeletal lesions 312 h after administration. CT, computed tomography. Gy, gray. h, hours. PET, positron emission tomography. SPECT, single-photon emission computed tomography. SUV, standardized uptake value.

**Figure 4:**
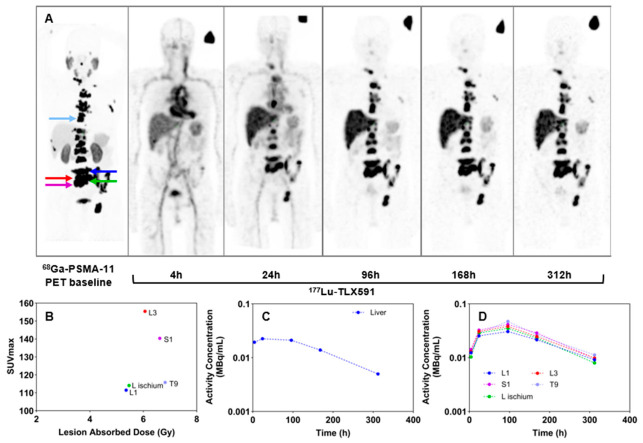
Representative patient images, SUV_max_ and activity concentration in liver and lesions. (**A**) Baseline ^68^Ga-PSMA-11 PET acquired at least 2 weeks prior to ^177^Lu-TLX591 therapy; lesions L1 (blue), left ischium (green), T9 (light blue), S1 (violet) and L3 (red) were used for dosimetry analysis. Serial SPECT maximum-intensity projections were acquired at 4, 24, 96, 168, and 312 h after the first ^177^Lu-TLX591 administration. (**B**) Descriptive plot of SUV_max_ with lesion dose (Gy). Activity concentration (MBq/mL) in liver (**C**) as the main organ of clearance and lesions (**D**), illustrating sustained activity in skeletal lesions through to 312 h (last protocol-specified imaging timepoint) after ^177^Lu-TLX591 administration. Gy, gray. h, hours. PET, positron emission tomography. SPECT, single-photon emission computed tomography. SUV, standardized uptake value.

Representative patient images showing SUV_max_ correlation with lesion dose and activity concentration in the liver (main clearance organ) and lesions are shown in Figure 3 and Figure 4.

### 3.4. Early Efficacy Signals

Of the patients in Cohort 2, 23 patients received both therapeutic doses of ^177^Lu-TLX591, of which 16 patients were included in the protocol-specified efficacy analysis (Figure 1). Among 16 evaluable patients, median rPFS was 8.8 months (95% CI, 4.0–11.3 months). One patient (6.3%) achieved PR, while thirteen (81.3%) patients had stable disease. At the time of trial termination, seven patients remained alive and progression-free at their last disease assessment.

Of the 23 patients in Cohort 2 who received both therapeutic doses of ^177^Lu-TLX591, 11 (47.8%; 95% CI, 26.8–69.4) patients experienced any PSA decline from baseline (Table 3). Six (26.1%; 95% CI, 10.2–48.4) patients experienced a ≥30% decrease, and three (13.0%; 95% CI, 2.8–33.6) patients experienced a ≥50% decrease. The mean (SD) duration of PSA30 and PSA50 responses from baseline was 167.0 ± 67.0 days and 139.7 ± 98.3 days, respectively. The median time to PSA30 and PSA50 responses was 29 and 43 days, respectively.

Of 16 patients, 6 (37.5%; 95% CI, 15.2–64.6) experienced any PSA response from baseline. Three (18.8%; 95% CI, 4.0–45.6) patients experienced a ≥30% decrease, and one (6.3%; 95% CI, 0.2–30.2) patient experienced a ≥50% decrease. The mean (SD) duration of PSA30 from baseline was 176.0 ± 61.2 days. The median time to PSA30 and PSA50 responses was 114 and 55 days, respectively (Table 3).

Of 23 patients in Cohort 2 who received both therapeutic doses of ^177^Lu-TLX591 and 16 patients of who met per-protocol criteria for inclusion in analysis, 20 (87.0%; 95% CI, 66.4–97.2) patients and 13 (81.3%; 95% CI, 54.4–96.0) patients, respectively, had any ALP response from baseline or any LDH response from baseline (Table 4).

**Figure 5:**
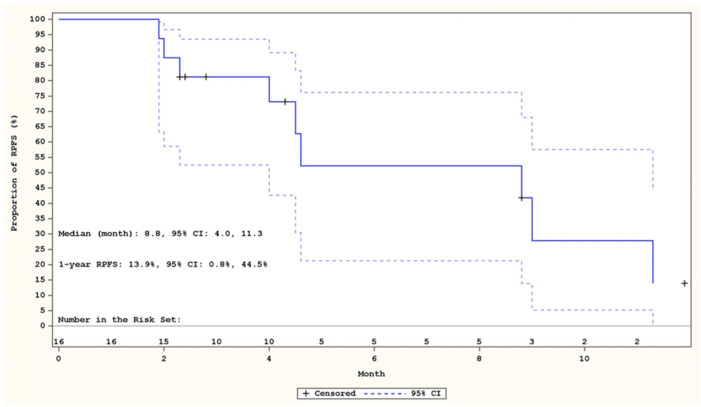
Radiographic progression-free survival, Kaplan–Meier estimates in evaluable patients from Cohort 2 (*n* = 16). rPFS was defined as the time from enrollment to radiographic disease progression or death from any cause, whichever occurred first. Dashed lines indicate 95% confidence intervals. Tick marks indicate censored observations. Median rPFS was 8.8 months (95% CI, 4.0–11.3 months). 1-year rPFS was 13.9% (95% CI, 0.8-44.5%).

A Kaplan–Meier estimate of PFS in Cohort 2 (*n* = 16) is shown in Figure 5. Radiographic progression-free survival was 8.8 months (95% CI, 4.0–11.3 months).

## 4. Discussion

The therapeutic landscape for patients with prostate cancer progressing on ARPIs remains an area of significant unmet need, particularly for patients with early metastatic disease that may have a more targetable disease and thus may derive greater benefit from therapy with a novel mechanism of action. In this context, ^177^Lu-TLX591 represents a differentiated PSMA-targeted approach that may address limitations of currently available RLTs, based on observed biodistribution characteristics. The results from ProstACT SELECT provide additional novel insight into the potential of ^177^Lu-TLX591 to demonstrate durable retention and limited off-target uptake in kidneys and salivary glands when administered in combination with SOC in a heterogenous patient sample with PSMA-avid disease confirmed on pre-therapy ^68^Ga-PSMA-11 PET imaging.

The aim of ProstACT SELECT was to evaluate patient selection for ^177^Lu-TLX591 therapy using ^68^Ga-PSMA-11 PET, in line with recommendations from regulatory agencies for imaging-based patient selection to confirm adequate presence of target prior to initiation of targeted therapy. A heterogenous patient sample was included, representing real-world clinical practice and best SOC as determined by investigator. Tumor targeting observed on ^177^Lu-TLX591 SPECT/CT imaging was consistent with uptake observed on ^68^Ga-PSMA-11 PET imaging, supporting utility of ^68^Ga-PSMA-11 PET to select patients appropriate for ^177^Lu-TLX591 therapy.

A notable finding of ProstACT SELECT was the persistence of measurable lesion-associated activity through the final protocol-specified imaging timepoint of 312 h (13.58 days) following administration of ^177^Lu-TLX591. The estimated lesion biological half-life of 326 h is consistent with the prolonged circulation and tumor residence characteristics expected of antibody-based PSMA-targeted agents. While imaging beyond 312 h was not performed, these data demonstrate sustained tumor-associated activity throughout the observation period. Whether prolonged retention translates into improved tumor control or clinical outcomes requires confirmation in adequately powered efficacy studies.

Absorbed radiation doses were consistent with the known biologic behavior of large monoclonal antibodies. Monoclonal antibodies generally exhibit minimal uptake in salivary glands because PSMA expression in these tissues is less accessible to circulating antibodies [23]. In this trial, only five patients experienced mild (Grade 1) xerostomia, which is consistent with the lack of salivary gland uptake observed on ^177^Lu-TLX591 SPECT/CT. Findings suggest the potential for low incidences of xerostomia, an adverse event that impacts quality of life and is commonly attributed to currently available small molecule-based PSMA-targeting RLTs [9]. Hepatic uptake was expected given the liver serves as the clearance organ, which is relatively radioresistant. No clinically meaningful hepatotoxicity was observed despite the liver receiving the highest absorbed organ dose. These findings are consistent with hepatic clearance serving as the primary elimination pathway for ^177^Lu-TLX591 and support the overall predictability of its biodistribution profile. Whether the biodistribution profile observed following ^177^Lu-TLX591 administrations translates into clinical benefit remains to be elucidated and is the basis for the ongoing Phase 3 ProstACT GLOBAL trial.

As PSMA-targeted RLTs move earlier in the disease continuum, limiting radiation exposure to at-risk organs is increasingly important. In this setting, preservation of critical organ functions, including renal, salivary gland, hepatic, and bone marrow functions, has implications for long-term safety and quality of life, as well as later treatment sequencing and eligibility for future therapies. Given the relatively low radiation exposure to critical organs, including kidneys and salivary glands, patients treated with ^177^Lu-TLX591 who later progress may plausibly be candidates for subsequent small-molecule RLT. Recently, a longitudinal analysis from the LUMEN registry demonstrated a statistically significant longitudinal decline in renal function in patients with mCRPC treated with ^177^Lu-PSMA-617 [15]. The rate of renal function loss accelerated with increasing cumulative administered activity, supporting a dose-dependent effect. Although this decline was considered unlikely to be clinically relevant in patients with late-stage mCRPC and limited life expectancy, the cumulative impact may become increasingly important as radiopharmaceutical therapies are applied earlier in the disease course, where patients may survive for many years following treatment. Even modest annual declines could accumulate into clinically meaningful reductions in renal reserve over time, particularly among patients receiving extended treatment courses.

The safety profile observed in ProstACT SELECT was generally consistent with expectations for rADC therapy. As previously reported with ^177^Lu-J591 [24,25], hematologic adverse events were the most prominent TEAEs, with thrombocytopenia and neutropenia representing the most common treatment-related adverse events. Grade 4 thrombocytopenia occurred in 20% of patients, while Grade 4 neutropenia, leukopenia, and lymphopenia occurred in 4% of patients each. All patients who experienced a Grade 4 event recovered; only one patient with Grade 4 thrombocytopenia required a platelet transfusion. These findings have potential clinical implications for treatment planning in patients treated with ^177^Lu-TLX591, as they suggest a predictable and monitorable hematologic safety profile when used alongside SOC. The observed reversibility of higher-grade hematologic events underlines the importance of routine laboratory surveillance and supportive care measures.

Limitations of the trial should be acknowledged. The enrolled population was heterogenous with respect to tumor burden, previous treatment regimen exposures, and SOC (determined by investigator) received while on trial. While this design reflects a real-world setting, differences in ongoing SOC treatments may have contributed to variability in clinical outcomes. In addition, efficacy analyses were performed in a protocol-defined efficacy population that excluded nine patients with important protocol deviations. Accordingly, the sample size included in the efficacy analysis was reduced; however, no formal sample size calculation was performed, and efficacy outcomes were intended for exploratory and indicative purposes only in the context of this Phase 1 trial. Reassuringly, biomarker response trends were generally similar between the per-protocol efficacy population and the broader population of patients who received both therapeutic doses of ^177^Lu-TLX591. Future studies comparing ^177^Lu-TLX591 in combination with SOC compared to SOC alone are warranted. Further interpretation is limited by the small sample size, single-arm design, and limited long-term follow-up of patients, all of which are consistent with Phase 1 trial designs. Thus, efficacy results should be interpreted with caution; a robust efficacy analysis is planned as part of the ongoing Phase 3 ProstACT Global trial.

## 5. Conclusions

^68^Ga-PSMA-11 PET-guided patient selection supported administration of the PSMA-targeting rADC ^177^Lu-TLX591 in patients with PSMA-expressing mCRPC. ^177^Lu-TLX591 demonstrated a manageable and predictable safety profile, sustained tumor-associated retention through the final imaging timepoint, low absorbed radiation dose to the salivary glands, and preliminary descriptive efficacy signals. Interpretation is limited by the small sample size, single-arm design, limited long-term follow-up, and exploratory efficacy analyses. These findings support further evaluation of ^177^Lu-TLX591 in larger, randomized studies powered for efficacy, including the ongoing Phase 3 ProstACT Global trial.

## Figures and Tables

**Table 1:** Patient demographics and prior medications for prostate cancer.

	Cohort 1 *n* = 5	Cohort 2 *n* = 25	Total *N* = 30
Age, median (SD; range)	68 (5.96; 59–73)	75 (7.37; 58–85)	72.5 (7.40; 58–85)
Race, *n* (%)			
Caucasian	5 (100)	18 (72.0)	23 (76.7)
Asian	0	1 (4.0)	1 (3.3)
Arabic	0	1 (4.0)	1 (3.3)
Not specific/reported	0	5 (20.0)	5 (16.7)
Ethnicity, *n* (%)			
Not Hispanic or Latino	5 (100)	20 (80.0)	25 (83.3)
Not reported	0	4 (16.0)	4 (13.3)
Unknown	0	1 (4.0)	1 (3.3)
Prior medications for prostate cancer *, *n* (%)			
Hormone antagonists and related agents	5 (100)	25 (100)	30 (100)
Hormones and related agents	5 (100)	18 (72.0)	23 (76.7)
Corticosteroids for systemic use	1 (20.0)	11 (44.0)	12 (40.0)
Bone protection agents	3 (60.0)	8 (32.0)	11 (36.7)
Plant alkaloids and other natural products (docetaxel)	2 (40.0)	5 (20.0)	7 (23.3)
Drugs used in benign prostatic hypertrophy	0	2 (8.0)	2 (6.7)

SD: standard deviation. Note: medications coded using the World Health Organization Drug Dictionary version 2021:09. * Any medication started before administration of ^177^Lu-TLX591, regardless of when medication was stopped.

**Table 2:** Mean absorbed radiation dose estimates by organ following administration of ^177^Lu-TLX591 (Gy/GBq).

Target Organ	Cohort 1 (Mean ± SD) *n* = 5	Cohort 2 (Mean ± SD) *n* = 25
Adrenals	0.142 ± 0.011	0.149 ± 0.01
Brain	0.080 ± 0.011	0.086 ± 0.01
Esophagus	0.101 ± 0.010	0.108 ± 0.01
Eyes	0.080 ± 0.011	0.086 ± 0.01
Gallbladder Wall	0.153 ± 0.013	0.163 ± 0.01
Left Colon	0.092 ± 0.010	0.098 ± 0.01
Small Intestine	0.091 ± 0.011	0.097 ± 0.01
Stomach Wall	0.100 ± 0.010	0.106 ± 0.01
Right Colon	0.100 ± 0.010	0.108 ± 0.01
Rectum	0.084 ± 0.011	0.091 ± 0.01
Heart Wall	0.104 ± 0.010	0.111 ± 0.01
Kidneys	0.676 ± 0.119	0.636 ± 0.17
Liver	2.316 ± 0.518	2.435 ± 0.56
Lungs	0.450 ± 0.075	0.445 ± 0.08
Pancreas	0.105 ± 0.009	0.113 ± 0.01
Prostate	0.084 ± 0.011	0.091 ± 0.01
Salivary Glands	0.078 ± 0.036	0.069 ± 0.03
Red Marrow	0.225 ± 0.184	0.168 ± 0.04
Osteogenic cells	0.167 ± 0.075	0.139 ± 0.03
Spleen	1.098 ± 0.666	0.988 ± 0.47
Testes	0.080 ± 0.012	0.087 ± 0.01
Thymus	0.090 ± 0.011	0.097 ± 0.01
Thyroid	0.086 ± 0.011	0.092 ± 0.01
Urinary Bladder Wall	0.083 ± 0.012	0.090 ± 0.01
Total Body	0.155 ± 0.014	0.161 ± 0.01

GBq, Gigabecquerel. Gy, gray. SD, standard deviation. Note: Organ dosimetry was derived from serial SPECT imaging and calculated using the MIRD schema and OLINDA/EXM software. Values are presented as mean ± SD (Gy/GBq).

**Table 3:** PSA responses and duration of response following ^177^Lu-TLX591 treatment in Cohort 2. Results are presented for all patients who received 2 therapeutic doses (*n* = 23) and for the protocol-defined efficacy analysis population (*n* = 16).

	Cohort 2 2.8 GBq × 2 Doses *n* = 23	Cohort 2 Efficacy Analyses * n = 16
Any PSA decline, *n* (%), 95% CI	11 (47.8), 26.8–69.4	6 (37.5), 15.2–64.6
PSA30		
≥30% decline, *n* (%), 95% CI	6 (26.1), 10.2–48.4	3 (18.8), 4.0–45.6
Mean duration, days ± SD	167.0 ± 73.4	176.0 ± 61.2
Median duration, days (min, max)	171.5 (54, 274)	170 (118, 240)
Median days to PSA30 response (min, max)	29.0 (28, 168)	114 (28, 168)
PSA50		
≥50% decline, *n* (%), 95% CI	3 (13.0), 2.8–33.6	1 (6.3), 0.2–30.2
Mean duration, days ± SD	139.7 ± 98.3	-
Median duration, days (min, max)	118.0 (54, 247)	118.0 (118, 118)
Median days to PSA50 response (min, max)	43 (29, 55)	55 (55, 55)

PSA: prostate-specific antigen. PSA30: reduction from baseline PSA level of at least 30%, maintained for at least 3 weeks. PSA50: reduction from baseline PSA level of at least 50%, maintained for at least 3 weeks. SD: standard deviation. Clopper–Pearson 95% CIs were calculated by Microsoft Copilot. * Patients who received 2.8 GBq × 2 doses (both therapeutic doses of ^177^Lu-TLX591) and met per-protocol criteria for inclusion in analysis.

**Table 4:** Changes in ALP and LDH levels following ^177^Lu-TLX591 treatment in Cohort 2. Results are shown for all patients who received 2 therapeutic doses (*n* = 23) and the protocol-defined efficacy analysis population (*n* = 16).

	Cohort 2 2.8 GBq × 2 Doses *n* = 23	Cohort 2 Efficacy Analyses * n = 16
Any ALP decline, *n* (%), 95% CI	20 (87.0), 66.4–97.2	13 (81.3), 54.4–96.0
Median change from baseline (min, max)	−15 (−98, 16)	−13 (−71, 16)
Any LDH decline, *n* (%), 95% CI	20 (87.0), 66.4–97.2	13 (81.3), 54.4–96.0
Median change from baseline (min, max)	−23 (−94, 13)	−23.5 (−94, 13)

ALP: alkaline phosphatase. GBq, Gigabecquerel. LDH: lactate dehydrogenase. * Patients who received 2.8 GBq × 2 doses (both therapeutic doses of ^177^Lu-TLX591) and met per-protocol criteria for inclusion in analysis. Clopper–Pearson 95% CIs were calculated by Microsoft Copilot.

## Data Availability

The de-identified patient dataset pertaining to results reported in this manuscript will be made available upon reasonable request after the intervention and indication are approved by both the FDA and EMA or 18 months after publication, whichever is latest.

## Supplementary Materials

The following supporting information can be downloaded at: https://www.mdpi.com/article/10.3390/cancers18142331/s1, Table S1: Protocol deviations leading to exclusions in efficacy analysis in Cohort 2; Table S2: Prior ARPIs and docetaxel therapy for prostate cancer (Cohort 2); Figure S1: Individual lesion absorbed dose following ^177^Lu-TLX591 therapy.

## Abbreviations

The following abbreviations are used in this manuscript:

ALP	Alkaline phosphatase
ARPI	Androgen receptor pathway inhibitor
CI	Confidence interval
CR	Complete response
CRPC	Castration-resistant prostate cancer
CT	Computed tomography
CTCAE	Common Terminology Criteria for Adverse Events
DES	Diethylstilbestrol
DICOM	Digital Imaging and Communications in Medicine
DSMB	Data safety monitoring board
ECG	Electrocardiogram
ECOG	Eastern Cooperative Oncology Group
EMA	European Medicines Agency
FDA	Food and Drug Administration
GBq	Gigabecquerel
Gy	Gray
h	Hour
IEC	Independent ethics committee
ICRP	International Commission on Radiological Protection
IRB	Institutional Review Board
LDH	Lactate dehydrogenase
mCi	Millicurie
mCRPC	Metastatic castration-resistant prostate cancer
MBq	Megabecquerel
MedDRA	Medical Dictionary for Regulatory Activities
min	Minute/minimum
MIRD	Medical internal radiation dose
MRI	Magnetic resonance imaging
ORR	Objective response rate
OS	Overall survival
OSEM	Ordered-subset expectation maximization
PCWG3	Prostate Cancer Working Group 3
PET	Positron emission tomography
PR	Partial response
PSA	Prostate-specific antigen
PSMA	Prostate-specific membrane antigen
rADC	Radio antibody–drug conjugate
RECIST	Response Evaluation Criteria in Solid Tumors
rPFS	Radiographic progression-free survival
RLT	Radioligand therapy
SD	Standard deviation
SOC	Standard of care
SPECT	Single-photon emission computed tomography
SSE	Symptomatic skeletal event
SUV	Standardized uptake value
SUVmax	Maximum standardized uptake value
Sv	Sievert
TEAE	Treatment-emergent adverse event
