# Supplementary material for: Theranostic Potential of 177Lu-TLX591 with Best Standard-of-Care and 68Ga-PSMA-11 PET for Patients with Metastatic Castration-Resistant Prostate Cancer: Results from the Phase 1 ProstACT SELECT Trial
Source: Cancers (Basel). 2026 Jul 20;18(14):2331. doi: 10.3390/cancers18142331 (PMC13406498; doi:10.3390/cancers18142331)
Supplement: Supplementary file 1 [file cancers-18-02331-s001.zip › cancers-4377829-supplementary.pdf]

Supplementary Table S1. Protocol deviations leading to exclusions in efficacy analysis in Cohort 2.

	Cohort 2 (n=25) n (%)
Eligibility criteria not met on retrospective review	4 (16)
Confirmatory scan not performed	4 (16)
Received ^{177}Lu-PSMA from another source	1 (4)

Note: The deviations listed above are those that, per the prespecified statistical analysis plan, resulted in exclusion from the per-protocol efficacy population.

Supplementary Table S2. Prior ARPIs and docetaxel therapy for prostate cancer (Cohort 2).

	n (%)
Prior docetaxel therapy	5 (20)
Prior ARPI therapy	
Abiraterone	7 (28)
Enzalutamide	7 (28)
ARPI doublet abiraterone + enzalutamide	2 (8)

ARPI, androgen receptor pathway inhibitor.

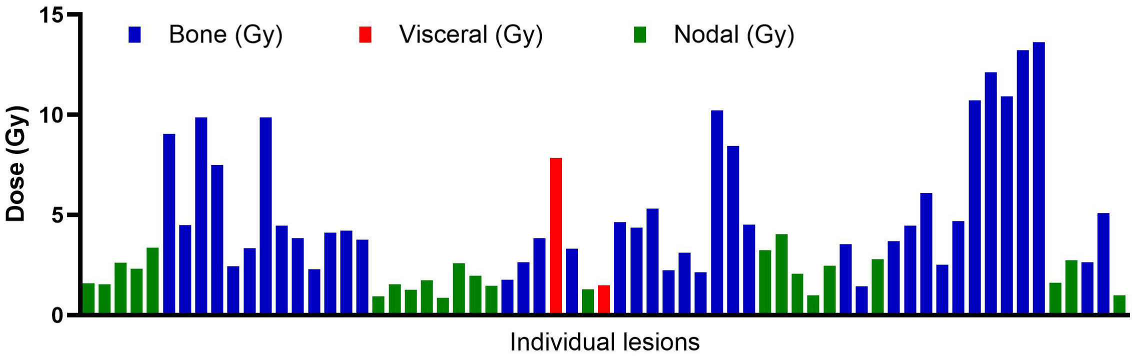

Supplementary Figure S1. Individual lesion absorbed dose following ^{177}Lu-TLX591 therapy. Absorbed dose estimates (Gy) for 65 lesions after 2 cycles of 2.8 GBq ^{177}Lu-TLX591. Bone lesions are shown in blue (n=40), nodal lesions in green (n=23), and visceral lesions in red (n=2).
